# Kaempferol Inhibits Endoplasmic Reticulum Stress-Associated Mucus Hypersecretion in Airway Epithelial Cells And Ovalbumin-Sensitized Mice

**DOI:** 10.1371/journal.pone.0143526

**Published:** 2015-11-24

**Authors:** Sin-Hye Park, Ju-Hyun Gong, Yean-Jung Choi, Min-Kyung Kang, Yun-Ho Kim, Young-Hee Kang

**Affiliations:** Department of Food and Nutrition, Hallym University, Chuncheon, Korea; Georgia Regents University, UNITED STATES

## Abstract

Mucus hypersecretion is an important pathological feature of chronic airway diseases, such as asthma and pulmonary diseases. MUC5AC is a major component of the mucus matrix forming family of mucins in the airways. The initiation of endoplasmic reticulum (ER)-mediated stress responses contributes to the pathogenesis of airway diseases. The present study investigated that ER stress was responsible for airway mucus production and this effect was blocked by the flavonoid kaempferol. Oral administration of ≥10 mg/kg kaempferol suppressed mucus secretion and goblet cell hyperplasia observed in the bronchial airway and lung of BALB/c mice sensitized with ovalbumin (OVA). TGF-β and tunicamycin promoted MUC5AC induction after 72 h in human bronchial airway epithelial BEAS-2B cells, which was dampened by 20 μM kaempferol. Kaempferol inhibited tunicamycin-induced ER stress of airway epithelial cells through disturbing the activation of the ER transmembrane sensor ATF6 and IRE1α. Additionally, this compound demoted the induction of ER chaperones such as GRP78 and HSP70 and the splicing of XBP-1 mRNA by tunicamycin. The *in vivo* study further revealed that kaempferol attenuated the induction of XBP-1 and IRE1α in epithelial tissues of OVA-challenged mice. TGF-β and tunicamycin induced TRAF2 with JNK activation and such induction was deterred by kaempferol. The inhibition of JNK activation encumbered the XBP-1 mRNA splicing and MUC5AC induction by tunicamycin and TGF-β. These results demonstrate that kaempferol alleviated asthmatic mucus hypersecretion through blocking bronchial epithelial ER stress via the inhibition of IRE1α-TRAF2-JNK activation. Therefore, kaempferol may be a potential therapeutic agent targeting mucus hypersecretion-associated pulmonary diseases.

## Introduction

Asthma is a long-term inflammatory disease of the airways characterized by recurring symptoms, reversible airflow obstruction and bronchospasm [1, 2]. The airway narrowing is caused by smooth muscle contraction and mucus hypersecretion, consequently resulting in asthmatic symptoms [3]. Chronic obstructive pulmonary disease (COPD) is also characterized by progressive airflow obstruction of the peripheral airways, associated with lung inflammation, emphysema and mucus hypersecretion [4, 5]. However, these pathological conditions of asthma and COPD may arise through directing different mechanisms at the cellular and molecular levels [6]. So far, there are no specific treatments for asthma and COPD that are considered as being effective in antagonizing the disease conditions. Nevertheless, there is a need to understand the pathophysiological mechanisms that could lead to new therapeutic strategies. Some of novel therapeutic approaches include the induction of goblet cell apoptosis, and the inhibition of mucin secretion and goblet cell hyperplasia [7].

The airway mucosa can respond to bacterial infection and allergic inflammation by surface mucous goblet cells, culminating in submucosal gland hyperplasia and hypertrophy with mucus hypersecretion [7, 8]. Airway mucus is acting as a physical and a biological fluid moved by the cilia. To understand the signaling pathways and mechanisms of mucin production and secretion have defined new therapeutic targets [9, 10]. The inhibition of Notch2 blocks goblet cell metaplasia, and Notch2 neutralization may be a therapeutic strategy for preventing basal cell differentiation toward a goblet cell fate in airway diseases [8]. Signaling of functional muscarinic receptors expressing from most notably epithelial cells and inflammatory cells regulates airway smooth muscle thickening and differentiation [11]. Potential targets for pharmacotherapy of hypersecretion in asthma have been identified [12]. Macrolide antibiotics as immunomodulatory medications possibly reduce mucin production as well as neutrophil migration by interfering with signal transduction of extracellular signal-regulated kinases [13]. Blocking transforming growth factor (TGF)-β effect inhibits epithelial shedding, mucus hypersecretion, airway smooth muscle cell hypertrophy and hyperplasia in an asthmatic mouse model [14]. Thus, reduction of TGF-β production and control of TGF-β effects would be a therapeutic intervention for airway remodeling in chronic asthma.

The endoplasmic reticulum (ER) is a specialized organelle that plays a central role in biosynthesis, correct protein folding, and posttranslational modifications of secretory and membrane proteins [15]. Dysfunction in ER homeostasis induces the ER stress response, resulting in unfolded protein response (UPR) activation [16, 17]. ER stress and the related signaling networks are emerging as important modulators in the development of allergen-induced severe bronchial asthma [18, 19]. These signaling pathways have been reported as crucial players in the pathogenesis of pulmonary disorders, including pulmonary fibrosis, lung injury, and COPD [20, 21]. One investigation has shown that ER stress mediates airway epithelial apoptosis and subepithelial fibrosis associated with loss of lung function [20]. The ER protein anterior gradient homolog 2 increases with overproduction of the mucins of 5AC (MUC5AC) and MUC5B in individuals with asthma and in mouse models of allergic airway disease [22]. However, the role of ER stress in the airway mucus production is not established.

**Fig 1:**
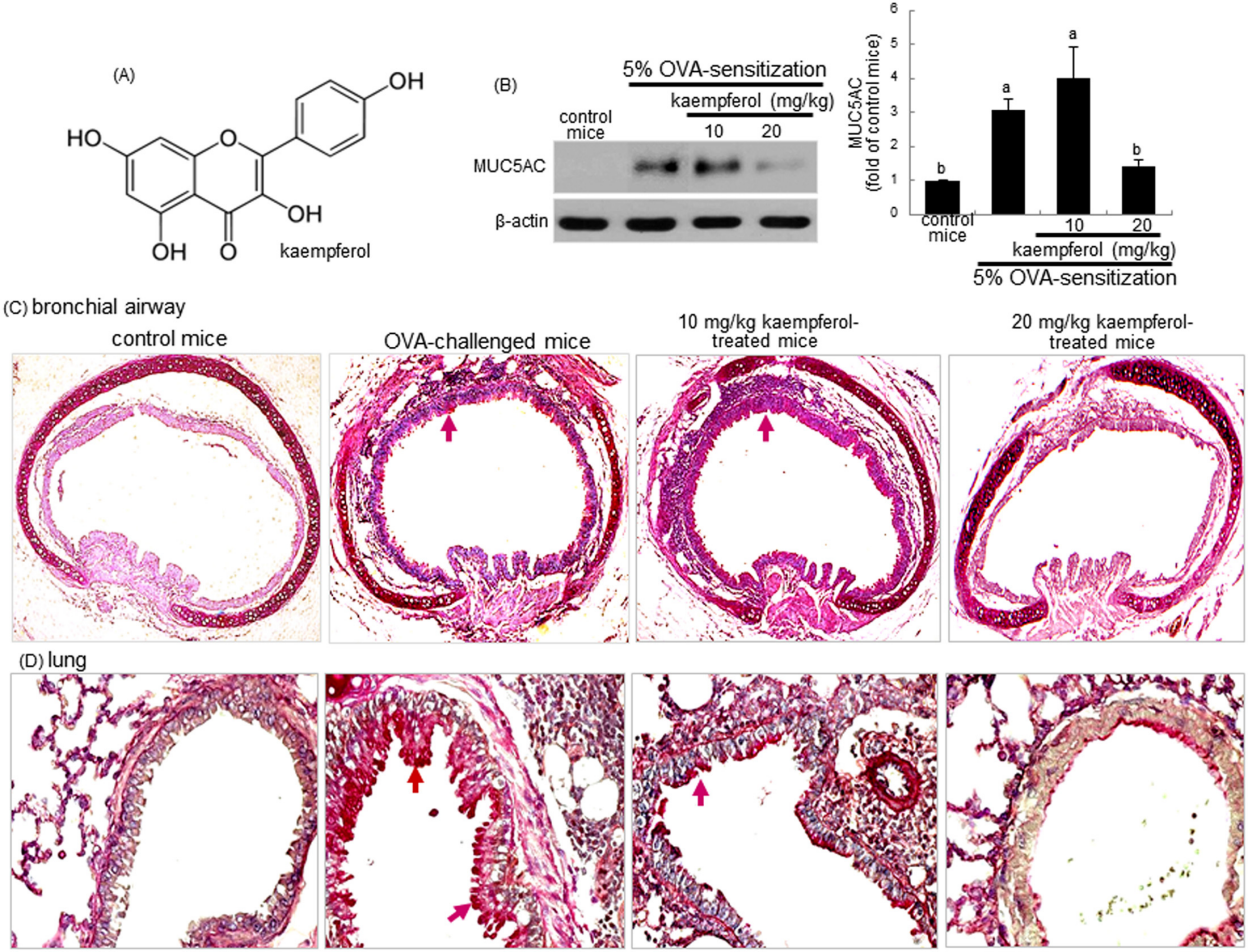
Chemical structure of kaempferol (A), and epithelial mucus hypersecretion in trachea and lung tissues (B). BALB/c mice were OVA-sensitized and orally supplemented with 10–20 mg/kg kaempferol. Lung tissue extracts were prepared for Western blot analysis with a primary antibody against MUC5AC. Representative blot data were obtained from 3 experiments, and β-actin protein was used as an internal control. The bar graphs (mean ± SEM) in the right panel represent quantitative results of blots. Values in bar graphs not sharing a letter indicate significant different at P<0.05. Tissue sections of mouse trachea and lung were stained by using PAS reagents and counterstained with hematoxylin (C and D). A strong PAS positive staining (red arrows) in many areas of mucous cells of airway epithelium was observed. Each photograph is representative of four mice.

Kaempferol (Fig 1A) is a polyphenol antioxidant abundant in berry fruits and vegetables [23]. Numerous studies have shown beneficial effects of dietary kaempferol in reducing the risk of chronic diseases by augmenting antioxidant defense against free radicals, and by modulating a number of key elements in cellular signaling pathways [23–25]. Kaempferol suppresses eosinophil infiltration, inflammation and fibrotic remodeling in airway epithelial cells and in mice with allergic asthma [26, 27]. However, the inhibitory effects of kaempferol on airway mucus hypersecretion in asthma are not well defined. This study investigated whether kaempferol encumbered airway production of MUC5AC by blocking ER stress in ovalbumin (OVA)-challenged mice. This study attempted to identify TGF-β signaling in ER stress as rational targets for treatment of excessive airway mucus production. Furthermore, the role of UPR was examined as a target for the therapeutic intervention of kaempferol in airway mucus production.

## Materials and Methods

### Chemicals

M199, human epidermal growth factor (EGF), hydrocortisone, gelatin, human insulin, apotransferrin, tunicamycin and albumin from chicken egg white were obtained from the Sigma-Aldrich Chemical (St. Louis, MO) as were all other reagents, unless specifically stated elsewhere. Fetal bovine serum (FBS), penicillin-streptomycin and trypsin-EDTA were purchased from the Lonza (Walkersville, MD). Human bronchial airway epithelial cell line BEAS-2B was provided by the American Type Culture Collection (Manassas, VA). Imject alum was purchased from Thermo Fisher Scientific (Rodkford, IL). Antibodies of human glucose-regulated protein (GRP)78, MUC5AC, X-box binding protein (XBP)-1, and phospho-inositol-requiring enzyme (IRE)1α were obtained from Abcam (Cambridge, UK). Antibodies against human tumor necrosis factor receptor associated factor (TRAF) 2, total c-Jun N-terminal kinase (JNK), and phospho-JNK (Thr183/Tyr185) were purchased from Cell Signaling Technology (Beverly, MA). Human heat shock protein (HSP) 70 antibody was purchased from the Santa Cruz Biotechnology (Santa Cruz, CA) and human β-actin antibody was obtained from Sigma-Aldrich Chemicals. The JNK inhibitor SP600125 was obtained from Calbiochem (Gibbstown, NJ). Horseradish peroxidase-conjugated goat anti-rabbit IgG, donkey anti-goat IgG and goat anti-mouse IgG were acquired from Jackson Immuno-Research Laboratories (West Grove, PA).

### BEAS-2B cell culture

Human lung bronchial epithelial BEAS-2B cells were cultured in 25 mM HEPES-buffered M199 containing 10% FBS, 2 mM L-glutamine, 100 U/ml penicillin, 100 μg/ml streptomycin supplemented with 2.5 μg/ml insulin, 0.361 μg/ml hydrocortisone, 2.5 μg/ml apo-transferrin and 20 ng/ml EGF. The 90–95% confluence of BEAS-2B cells was sustained at 37°C in an atmosphere of 5% CO_2_ during cell culture experiments. To induce airway mucus hypersecretion and ER stress, BEAS-2B cells were seeded at 80% confluence a day prior to starting the treatment of 1–20 μM kaempferol on 6-well or 12-well plates, and then stimulated with tunicamycin and TGF-β for 24–72 h.

### Induction of airway fibrosis in a murine model

Six week-old male BALB/c mice (Hallym University Breeding Center for Laboratory Animals) were used in the present study. Mice were kept on a 12 h light/12 h dark cycle at 23 ± 1°C with 50 ± 10% relative humidity under specific pathogen-free conditions, fed a non-purified diet (RodFeed^™^, DBL, Umsung, Korea) and were provided with water ad libitum at the animal facility of Hallym University. The non-purified diet composition was as follows: NLT (Not Less Than) 20.5% crude protein, NLT 3.5% crude fat, NMT (Not More Than) 8.0% crude fiber, NMT 8.0% crude ash, NLT 0.5% calcium and NLT 0.5% phosphorus. Mice were allowed to acclimatize for 1 week before beginning the experiments. Mice were divided into four subgroups (n = 6 for each subgroup). Mice were sensitized with 20 μg OVA dissolved in a solution of 30 μl phosphate buffered saline (PBS) and 50 μl Imject Alum by subcutaneous injection twice on d 0 and d 14. For dietary interventions, 0.1 ml kaempferol solution (10 or 20 mg/kg BW) was orally administrated to OVA-sensitized mice 1 h before challenge. On the day 28, 29 and 30, 5% OVA inhalation to mice was carried out for 20 min in a plastic chamber linked to an ultrasonic nebulizer (Clenny^2^ Aerosol, Medel, S. Polo di Torrile, Italy). Control mice were sensitized and challenged with PBS as the OVA vehicle. All mice were sacrificed with an anesthetic (2 μl/kg rompun and 8 μl/kg zoletil, intraperitoneal injection) 24 h after the last challenge (day 30). The right lungs were collected, frozen to liquid nitrogen and kept at -80°C for the extraction and the left lungs were preserved and fixed in 4% paraformaldehyde and then used for the staining.

All experiments were approved by the Committee on Animal Experimentation of Hallym University and performed in compliance with the University's Guidelines for the Care and Use of Laboratory Animals (Hallym 2010–66). No mice were dead and no apparent signs of exhaustion were observed during the experimental period.

### Western blot analysis

BALB/c lung tissue extracts or whole BEAS-2B cell lysates or were prepared in 1 mM Tris-HCl (pH 6.8) lysis buffer containing 10% SDS, 1% glycerophosphate, 0.1 mM Na_3_VO_4_, 0.5 mM NaF and protease inhibitor cocktail. Equal amounts of proteins in cell lysates or tissue extracts were electrophoresed on 8–15% SDS-PAGE and transferred onto a nitrocellulose membrane. Blocking to avoid a non-specific binding was performed using either 3% fatty acid-free BSA buffer or 5% non-fat dry milk for 3 h. The membrane was incubated overnight at 4°C with a specific primary antibody of MUC5AC, GRP78, XBP-1, HSP70, total JNK, phospho-JNK, phospho-IRE1α or TRAF2. The membrane was then applied to a secondary antibody of goat anti-rabbit IgG or goat anti-mouse IgG conjugated to horseradish peroxidase for 1 h. Following another triple washing, the target protein was determined using the Supersignal West Pico Chemiluminescence detection reagents (Pierce Biotechnology, Rockford, IL) and the Agfa medical X-ray film blue (Agfa HealthCare NV, Mortsel, Belgium). Incubation with mouse anti-human β-actin antibody was conducted for the comparative control.

### Periodic acid-Schiff (PAS) staining

For the histological analyses, lung specimens were obtained at the end of the experiments and fixed in 10% buffered formalin. The paraffin-embedded lung specimens were sectioned at 5 μm thickness, de-paraffinized and stained with PAS stain to assess goblet cell hyperplasia as a measure of airway mucus hypersecretion. The stained tissue sections were examined using an optical microscope AXIOIMAGER (Zeiss, Gottingen, Germany), and five images were taken for each section.

### RT-PCR analysis

Total RNA was obtained from BEAS-2B cells (a density of 6 × 10^5^/60mm dish) using a commercial Trizol reagent kit (Invitrogen, Carlsbad, CA), and cDNA was synthesized using 5 μg total RNA with 0.5 ng/ml oligo-(dT)_15_ primer (Bioneer, Daejeon, Korea) and 200 units of reverse transcriptase. The PCR (Bio-Rad Laboratories, Hercules, CA) was accomplished using primers including human spliced XBP1 (forward primer: 5’-TACGGGAGAAAACTCACGGC-3’, reverse primer: 5’-TTCCAGCTTGGCTGATGAGG-3’, 421 bp) and glyceraldehyde-3-phosphate dehydrogenase (GAPDH, forward primer: 5’-CCTCCTGTTCGACAGTCAGC-3’, reverse primer: 5’-CGCCCAATACGACCAAATCC-3’, 113 bp) with an addition of 25 μl of 10 mM Tris-HCl (pH 9.0) containing 25 mM MgCl_2_, 10 mM dNTP and 5 units of Taq DNA polymerase. The PCR reaction was performed under the condition of 30 s denaturation at 94°C, 45 s annealing at 60°C and 45 s elongation at 72°C as a cycle. After thermocycling, electrophoresis on 3% agarose-formaldehyde gel containing 0.5 μg/ml ethidium bromide was achieved, and bands were visualized, taken and quantified.

### Immunohistochemical staining

For the immunohistochemical analysis, paraffin-embedded trachea tissue sections (5 μm thick) were employed. The sections were placed on glass slides, de-paraffinated and hydrated with xylene and graded alcohol. The sections were pre-incubated in a boiling sodium citrate buffer (10 mM sodium citrate, 0.05% Tween 20, pH 6.0) for antigen retrieval. Specific primary antibody against mouse XBP1 was incubated with the tissue sections overnight. Subsequently, the tissue sections were incubated for 1 h with Cy3-conjugated anti-rabbit IgG. Nuclear staining was done with 4',6-diamidino-2-phenylindole (DAPI). For the detection of for phospho-IRE1α and TRAF2 in lung tissues, chromogenic substrate detection kits (Dako, Carpinteria, CA) of 3,3'-diaminobenzidine (DAB) and 3-amino-9-ethylcarbazol (ACE) were used, respectively. Counter-staining was conducted with hematoxylin. Each slide was mounted in VectaMount mounting medium (Vector Laboratories, Burlingame, CA). Images of each slide were taken using an optical microscope system (Axiomager, Zeiss, Germany).

### Statistical analysis

The results were expressed as mean ± SEM for each treatment group in the *in vivo* and *in vitro* experiments. Statistical analyses were performed using Statistical Analysis Systems statistical software package (SAS Institute, Cary, NC). Significance was determined by one-way ANOVA, followed by Duncan range test for multiple comparisons. Differences were considered significant at P<0.05.

## Results

### Inhibition of mucus hypersecretion and goblet cell hyperplasia by kaempferol

Goblet cell hyperplasia and mucus hypersecretion were observed in bronchial asthma [28]. The present study evaluated the inhibitory effects of kaempferol on the mucus hypersecretion in bronchial airway and lung tissues in OVA- experienced mice. The MUC5AC gene is the major inducible mucus gene in the lung airway epithelium and is linked to mucus hypersecretion in the pulmonary tracts and associated to COPD [29]. As shown in Fig 1B, the MUC5AC protein was highly induced in OVA-exposed lung tissues, which was blocked by administrating 20 mg/kg kaempferol to mice. Consistently, there was a strong red staining in the both tissue sections of OVA- experienced mice observed, as evidenced by PAS staining (Fig 1C and 1D, arrows). However, in OVA-experienced mice supplemented with 20 mg/kg kaempferol, the staining was markedly diminished, indicating that this compound inhibited the OVA-triggered mucus production (Fig 1C and 1D).

**Fig 2:**
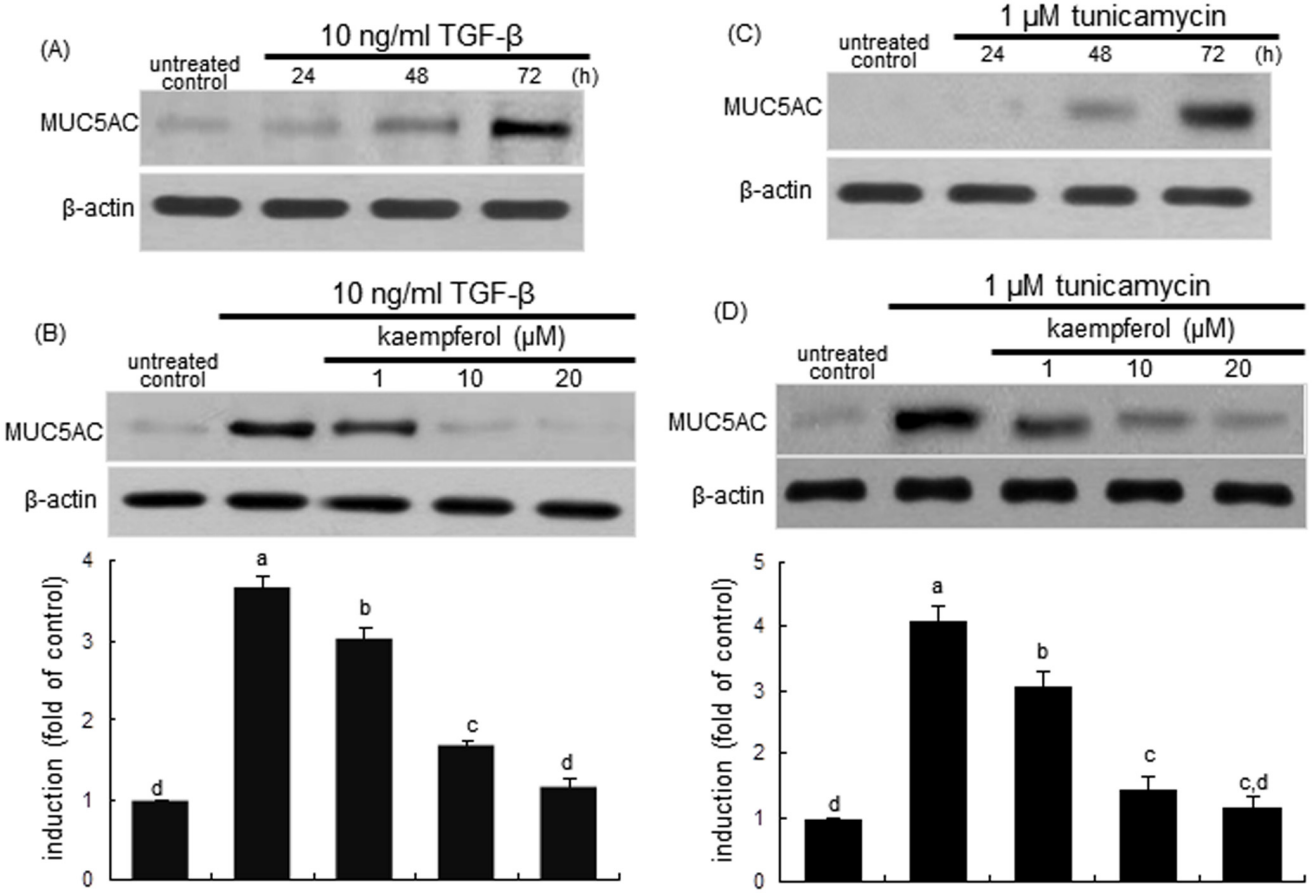
Time course responses of MUC5AC induction to TGF-β and tunicamycin (A and C), and inhibition of MUC5AC induction by kaempferol (B and D). BEAS-2B cells were treated with 10 ng/ml TGF-β or 1 μM tunicamycin up to 72 h in the absence and presence of 1–20 μM kaempferol. For the measurement of MUC5AC induction, total cell lysates were subject to Western blot analysis with a primary antibody against MUC5AC. β-Actin was used as an internal control. The bar graphs (mean ± SEM) in the bottom panels represent quantitative results of blots. Values not sharing a common letter are significantly different at P<0.05.

This study determined whether the profibrotic cytokine TGF-β promoted cellular over-expression of the MUC5AC protein, which was demoted by kaempferol. TGF-β initiated MUC5AC induction from 48 h after its exposure, and the induction was highly elevated at 72 h (Fig 2A). Kaempferol at the doses of ≥1 μM dose-dependently diminished the elevated induction of MUC5AC in TGF-β-exposed BEAS-2B cells for 72 (Fig 2B). Similarly, the marked up-regulation of MUC5AC was observed at 72 h after the exposure of airway epithelial cells to the ER stressor tunicamycin (Fig 2C), which was significantly dampened by ≥1 μM kaempferol (Fig 2D). Accordingly, both TGF-β and tunicamycin increased cellular expression of MUC5AC from airway epithelial BEAS-2B cells. It can be deemed that ER stress may cause mucus hypersecretion in airways, which would be reversed by kaempferol.

### Blockade of ER stress and UPR activation by kaempferol

**Fig 3:**
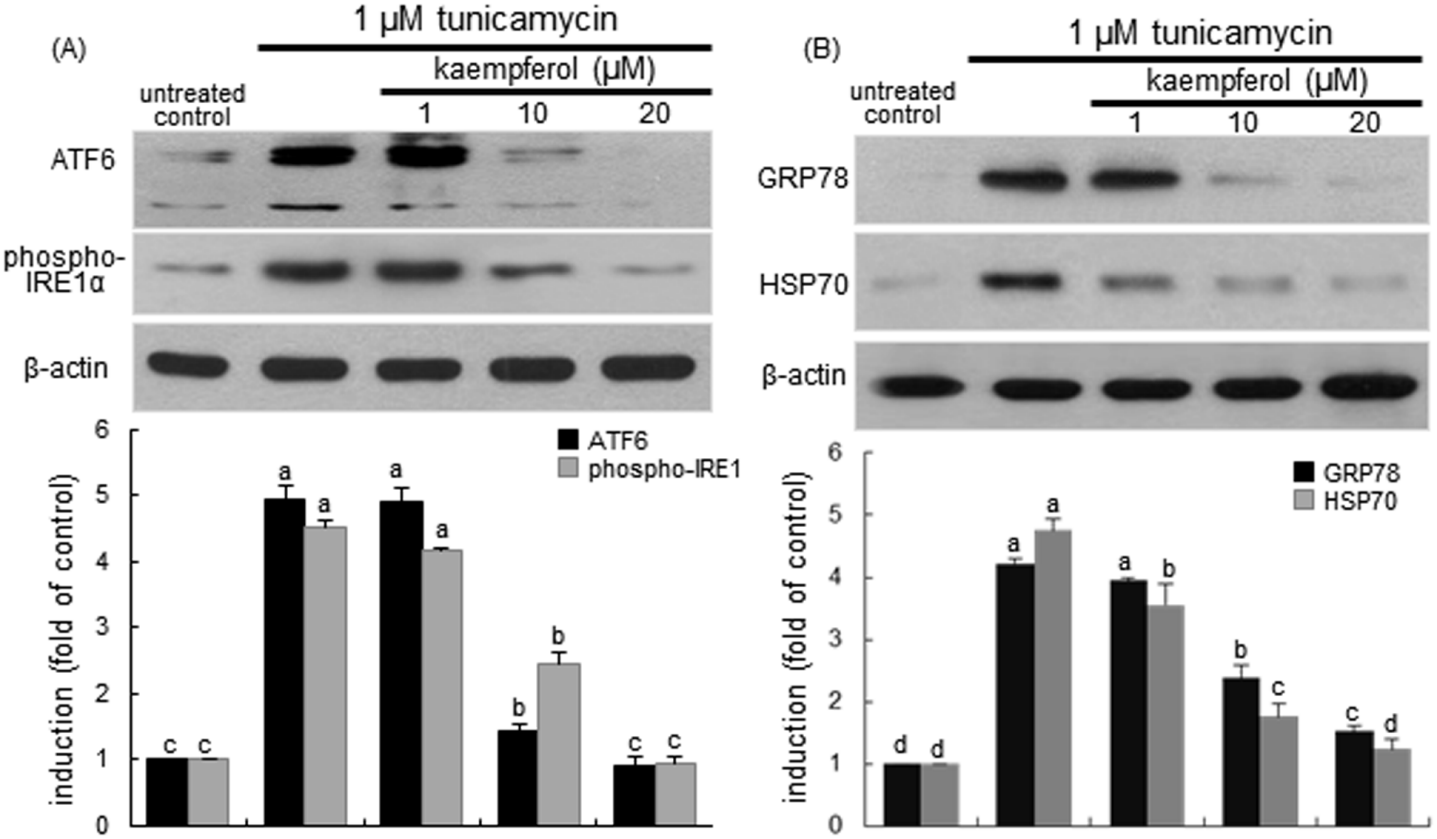
Inhibitory effects of kaempferol on tunicamycin-induced expression and activation of ATF6 and IRE1α (A), and GRP78 and HSP70 (B) in BEAS-2B cells. Cells were treated with 1–20 μM kaempferol and simulated with 1 μM tunicamycin. Cell lysates were prepared for Western blot analysis with a primary antibody against ATF6, phospho-IRE1α, GRP78 and HSP70. Representative blot data were obtained from 3 experiments, and β-actin protein was used as an internal control. The bar graphs (mean ± SEM) in the bottom panels represent quantitative results of upper blots. Values in bar graphs not sharing a letter indicate significant different at P<0.05.

This study investigated whether kaempferol suppressed ER stress or UPR in the airway epithelium. When airway BEAS-2B cells were exposed to 1 μM tunicamycin for 24 h, the specific UPR signaling components, such as ER transmembrane transcription factor ATF6 and IRE1α were greatly induced and activated (Fig 3A). The induction of ATF6 and IRE1α by treating tunicamycin was dose-dependently suppressed by ≥10 μM kaempferol. In addition, the 24 h-treatment of tunicamycin stimulated the induction of ER molecular chaperones of GRP78 and HSP70 (Fig 3B). In contrast, ≥10 μM kaempferol inhibited the epithelial induction of GRP78. Also, 1–20 μM kaempferol encumbered such elevated induction of HSP70 in a dose-dependent manner (Fig 3B). Accordingly, kaempferol may ameliorate ER stress via retarding the activation of the UPR components of ATF6, IRE1α, GRP78 and HSP70.

**Fig 4:**
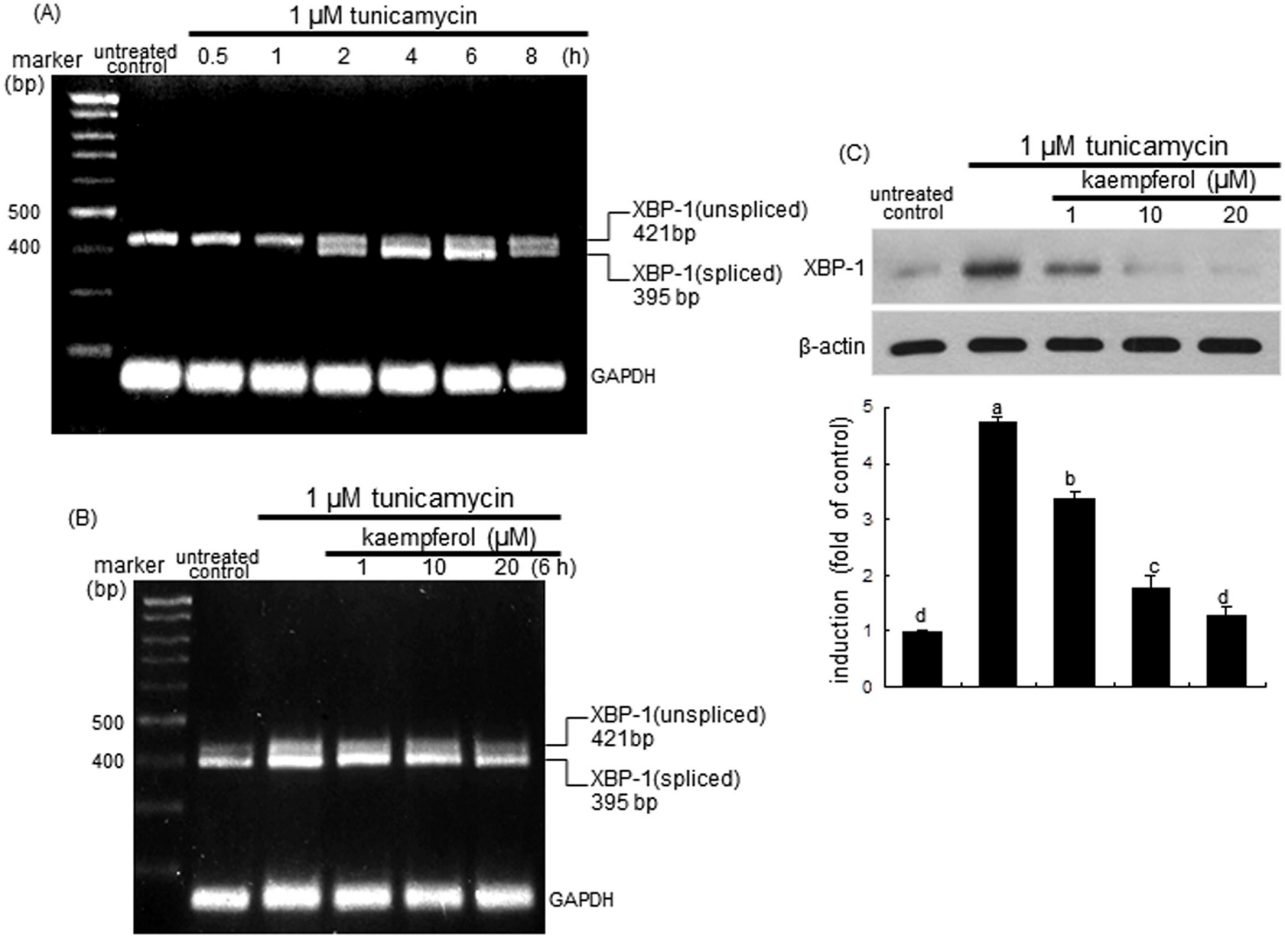
RT-PCR data showing XBP-1 mRNA splicing (A and B) and Western blot data showing XBP-1 induction (C) in tunicamycin-stimulated and kaempferol-supplemented BEAS-2B cells. Cells were treated with 1–20 μM kaempferol and simulated with 1 μM tunicamycin. GAPDH was used as a housekeeping gene for the co-amplification with XBP-1 (A and B). Cell lysates were prepared for Western blot analysis with a primary antibody against XBP-1 (C). Representative blot data were obtained from 3 experiments, and β-actin protein was used as an internal control. The bar graphs (mean ± SEM) in the bottom panel represent quantitative results of upper blots. Values in bar graphs not sharing a letter indicate significant different at P<0.05.

The XBP-1 mRNA splicing of airway epithelial BEAS-2B cells was induced from 2 h after ER insult by tunicamycin, and such induction was sustained for another 2 h (Fig 4A). The XBP-1 mRNA splicing was noticeably observed from 4 h after the exposure to tunicamycin. In contrast, the splicing of XBP-1 mRNA was dose-dependently demoted by treating BEAS-2B cells with 1–20 μM kaempferol (Fig 4B). Consistently, Western blot analysis showed that the XBP-1 protein induction was greatly elevated in tunicamycin-stimulated BEAS-2B cells, which was reversed by treating 1–20 μM kaempferol (Fig 4C).

### Kaempferol inhibition of ER stress induced by TGF-β

**Fig 5:**
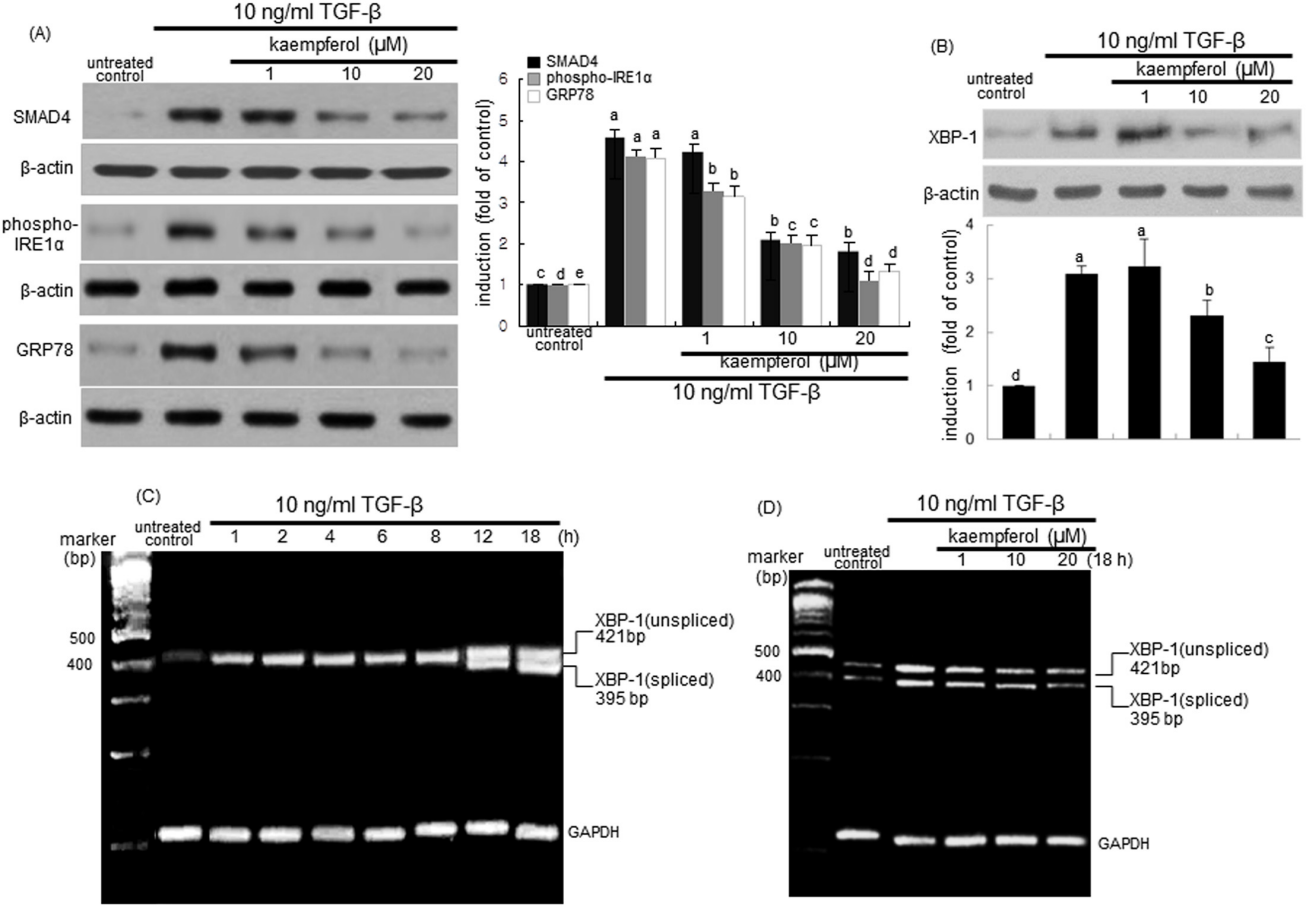
Suppressive effects of kaempferol on induction and activation of SMAD4, IRE1α and GRP78 (A), and XBP-1 protein induction (B) and XBP-1 mRNA transcription and splicing (C and D) in TGF-β-stimulated BEAS-2B cells. Cells were treated with 1–20 μM kaempferol and simulated with 10 ng/ml TGF-β. Cell lysates were prepared for Western blot analysis with a primary antibody against SMAD4, phospho-IRE1α, GRP78 and XBP-1. Representative blot data were obtained from 3 experiments, and β-actin protein was used as an internal control. The bar graphs (mean ± SEM) represent quantitative results of blots. Values in bar graphs not sharing a letter indicate significant different at P<0.05. RT-PCR was conducted to determine XBP-1 mRNA splicing (C and D). GAPDH was used as a housekeeping gene for the co-amplification with XBP-1.

The ER stressor tunicamycin increased cellular expression of MUC5AC from airway epithelial BEAS-2B cells (Fig 2C). It is assumed that ER stress may cause airway epithelial mucus hypersecretion. Similarly, TGF-β increased epithelial induction of MUC5AC in airways (Fig 2A). Accordingly, this study determined whether TGF-β could trigger ER stress, which would be inhibited by kaempferol. When 10 ng/ml TGF-β was added to airway epithelial cells for 24 h, the induction of IRE1α and GRP78 was markedly enhanced (Fig 5A). It should be noted that the SMAD4 expression was significantly elevated by TGF-β, indicating that this cytokine-SMAD4 signaling may be involved in the ER stress of airway epithelial cells. Submicromolar kaempferol blocked epithelial induction of IRE1α and GRP78 by TGF-β concomitantly with the inhibition of the SMAD4 expression (Fig 5A). Western blot analysis showed that TGF-β-stimulated XBP-1 protein induction was reversed by treating ≥10 μM kaempferol to BEAS-2B cells (Fig 5B). Furthermore, the XBP-1 mRNA transcription and splicing were markedly promoted from 12 h after TGF-β stimulation (Fig 5C). Such XBP-1 mRNA splicing elevated by the 18 h-TGF-β stimulation was substantially blunted in BEAS-2B cells treated with kaempferol (Fig 5D).

### Inhibition of OVA-challenged UPR induction by kaempferol

**Fig 6:**
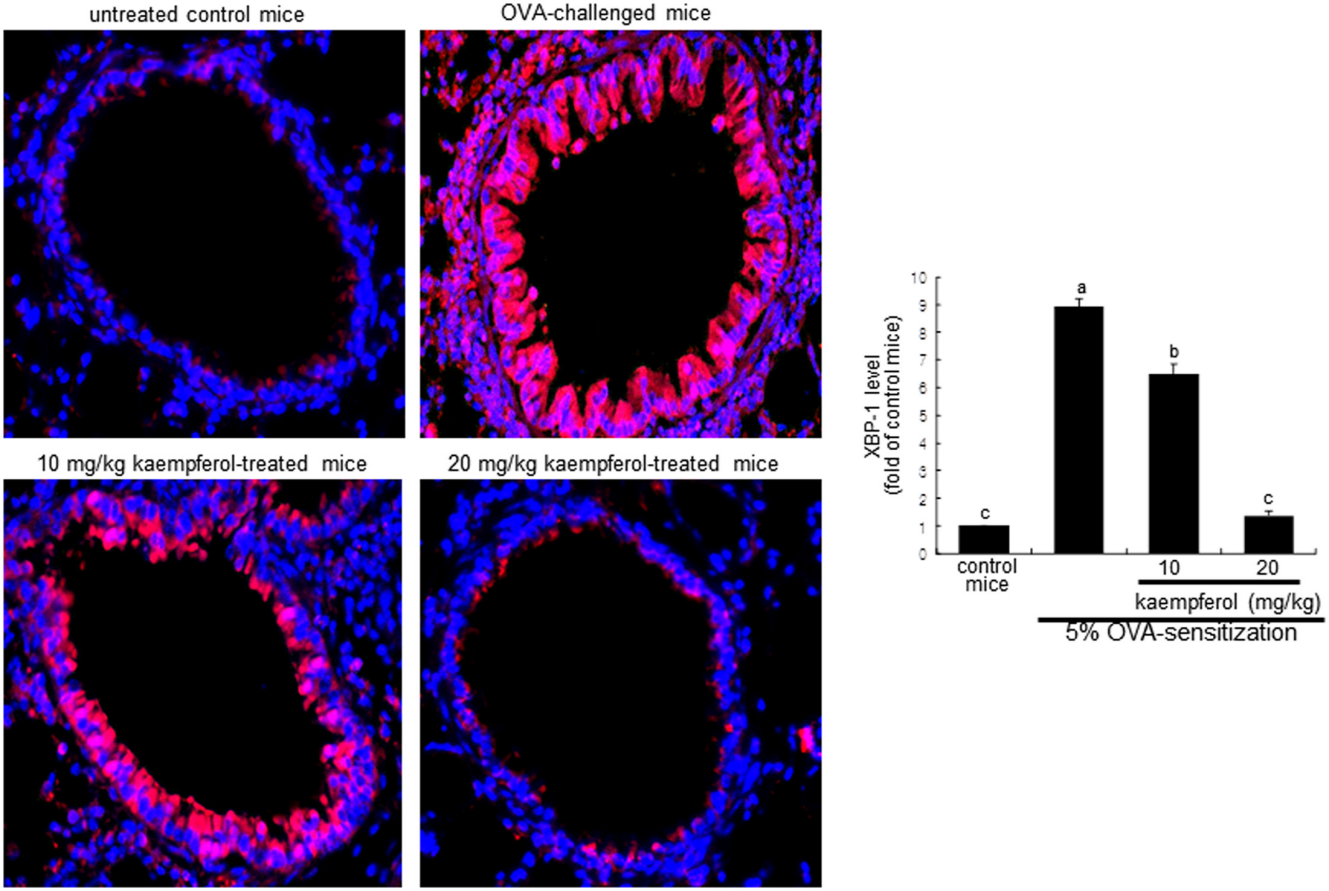
Immunofluorescent data showing inhibition of XBP-1 induction in OVA-challenged mouse lung tissues by kaempferol. Epithelial XBP-1 protein was identified as reddish and/or pinkish staining. XBP-1 was visualized with a Cy3-conjugated secondary antibody and nuclear staining was done with DAPI. Each photograph is representative of four mice. Magnification: 200-fold. The bar graphs (mean ± SEM) represent quantitative results of Cy3 staining. Values in bar graphs not sharing a letter indicate significant different at P<0.05.

**Fig 7:**
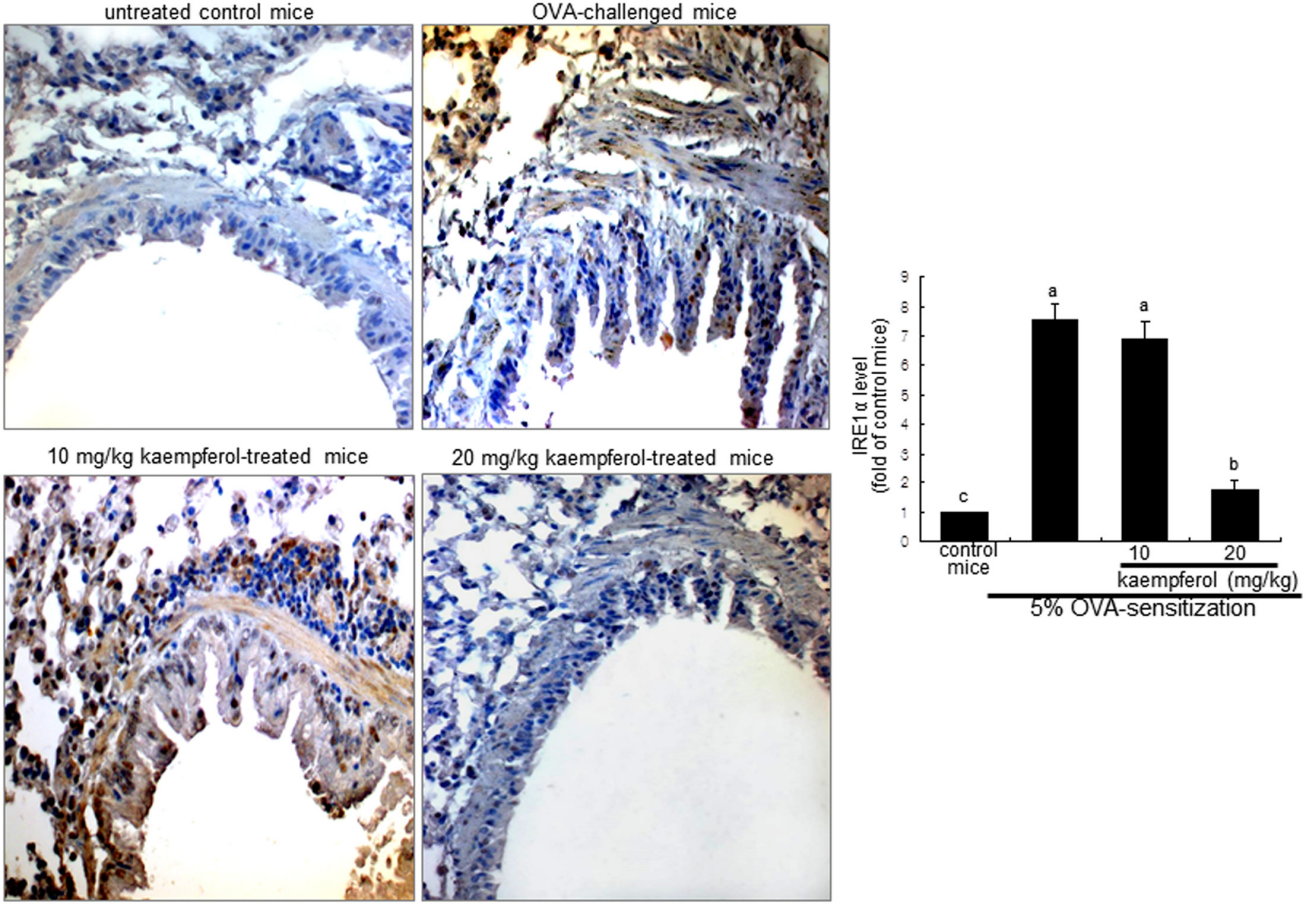
Immunohistochemical staining showing inhibition of IRE1α activation in OVA-challenged mouse lung tissues by kaempferol. Epithelial IRE1α was identified as brown staining and was visualized with DAB and the counter-staining was done with hematoxylin. Each photograph is representative of four mice. Magnification: 200-fold. The bar graphs (mean ± SEM) represent quantitative results of DAB staining. Values in bar graphs not sharing a letter indicate significant different at P<0.05.

This study examined the XBP-1 induction in lung tissues of OVA-challenged mice, as evidenced by immunofluorohistochemical staining. As expected, there was lack of airway epithelial staining in the negative control mice (Fig 6). However, a strong reddish staining was observed in OVA-challenged mouse airways. In contrast, the lung tissue levels of XBP-1 declined dose-dependently in mice supplemented with ≥10 mg/kg kaempferol (Fig 5). In addition, the OVA challenge to sensitized mice enhanced lung tissue IRE1α (brown staining) induced by misfolded proteins in the lumen of ER, while oral administration of 20 mg/kg kaempferol demoted such induction (Fig 7).

### Disruption of IRE1α-TRAF2-JNK signaling by kaempferol

**Fig 8:**
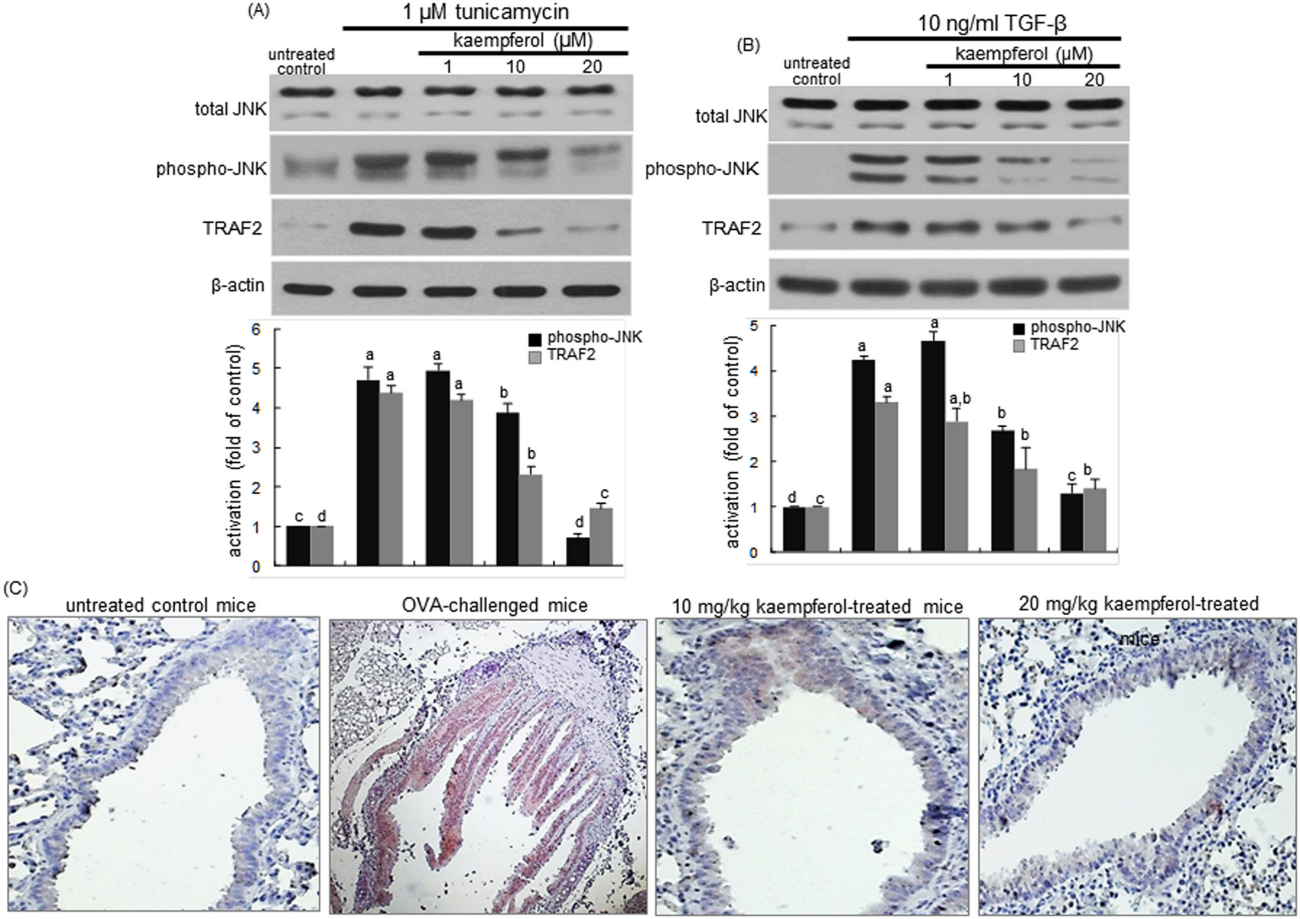
Inhibition of JNK activation and TRAF2 induction in BEAS-2B cells treated with kaempferol and exposed to 1 μM tunicamycin (A) or 10 ng/ml TGF-β (B). Cell extracts were subject to 12% SDS-PAGE and western blot analysis with a primary antibody against total JNK, phospho-JNK and TRAF2. β-Actin protein was used as an internal control. The bar graphs (mean ± SEM) in the bottom panels represent quantitative results of blots obtained from a densitometer. Values not sharing a common letter are significantly different at P<0.05. BALB/c mice were OVA-sensitized and orally supplemented with 10–20 mg/kg kaempferol (C). Epithelial TRAF2 was identified as reddish staining and was visualized with ACE and the counter-staining was done with hematoxylin. Each photograph is representative of four mice. Magnification: 200-fold.

This study examined that kaempferol inhibited the IRE1α-TRAF2 activation by the ER stressor of tunicamycin and TGF-β in airway epithelial cells. When airway epithelial cells were treated with 1 μM tunicamycin for 24 h, there were marked JNK activation and TRAF2 induction observed (Fig 8A). Similarly, the JNK activation and TRAF2 induction occurred in TGF-β-exposed epithelial cells (Fig 8B). In contrast, the presence of kaempferol dampened such activation and induction in ER stress-experiencing epithelial cells by tunicamycin and TGF-β (Fig 8A and 8B). Moreover, the lung tissue TRAF2 was obviously induced in OVA-challenged mice and oral administration of ≥10 mg/kg kaempferol prevented such induction (Fig 8C).

**Fig 9:**
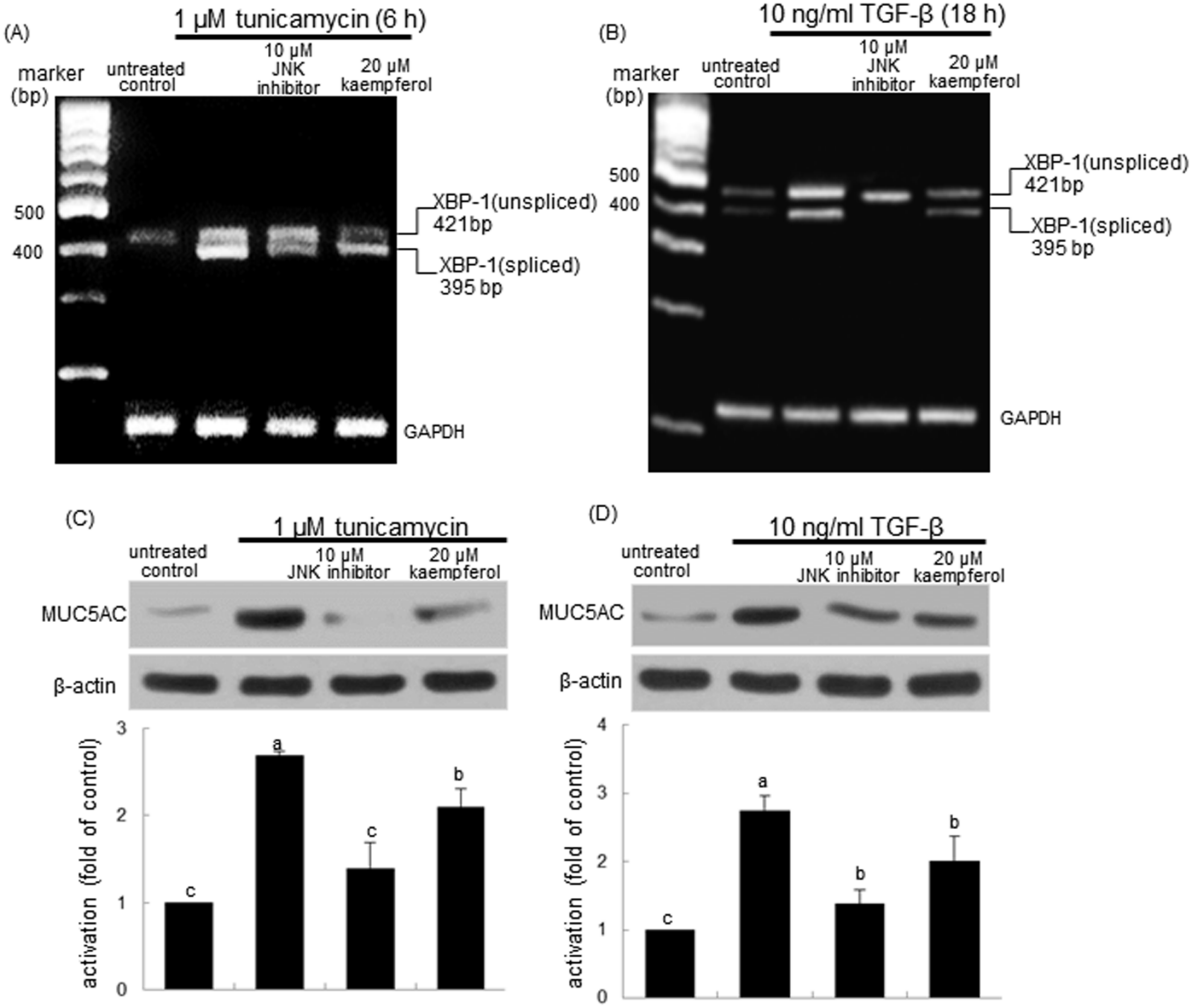
Inhibition of XBP-1 mRNA splicing (A and B) and MUC5AC induction (C and D) by JNK inhibitor. BEAS-2B cells were treated with kaempferol and exposed to 1 μM tunicamycin or 10 ng/ml TGF-β. RT-PCR was conducted to determine XBP-1 mRNA splicing (A and B). GAPDH was used as a housekeeping gene for the co-amplification with XBP-1. Cell extracts were subject to 12% SDS-PAGE and Western blot analysis with a primary antibody against MUC5AC. β-Actin protein was used as an internal control. The bar graphs (mean ± SEM) in the bottom panels represent quantitative results of blots obtained from a densitometer. Values not sharing a common letter are significantly different at P<0.05.

The induction and signaling of IRE1 across ER lead to JNK activation dependent on TRAF2 that binds to the cytoplasmic region of IRE1 [30]. This study investigated that kaempferol interfered with the IRE1α-TRAF2 dependence of the JNK pathway. As seen with 20 μM kaempferol, the inhibition of JNK activation by the JNK inhibitor dampened the XBP-1 splicing of airway epithelial cells in the presence of tunicamycin and TGF-β (Fig 9A). Similarly, the JNK inhibitor notably blocked the XBP-1 mRNA transcription and splicing by TGF-β (Fig 9B). Furthermore, like 20 μM kaempferol the JNK inhibition resulted in retarding the MUC6AC induction by tunicamycin and TGF-β (Fig 9C and 9D).

## Discussion

Seven major findings were extracted from this study. 1) Oral administration of kaempferol reduced lung tissue levels of MUC5AC and alleviated airway goblet cell hyperplasia in OVA-challenged mice. 2) The MUC5AC protein was induced by the exposure to both TGF-β and tunicamycin for 72 h, which was dampened by ≥1 μM kaempferol. 3) Kaempferol deterred ER stress through diminishing the 24 h-induction and activation of UPR components of ATF6 and IRE-1α and chaperones of GRP78 and HSP70. 4) Tunicamycin-induced XBP-1 mRNA splicing were encumbered by adding kaempferol to airway epithelial cells. 5) TGF-β induced ER stress concomitant with the splicing of XBP-1 mRNA and the induction of the UPR components, while kaempferol attenuated the TGF-β-triggered ER stress. 6) The supplementation of kaempferol reduced the tissue levels of XBP-1, IRE-1α and TRAF2 in the goblet cells and epithelium of OVA-challenged mouse airways. 7) Kaempferol blunted the JNK activation and TRAF2 induction by tunicamycin and TGF-β, and the inhibition of JNK activation attenuated the XBP-1 splicing and MUC5AC induction. Accordingly, kaempferol alleviated allergen-induced mucus hypersecretion through blunting bronchial epithelial ER stress linked to IRE-1α-TRAF2-JNK triggered by TGF-β signaling.

The flavonol-type polyphenol kaempferol has been reported to reduce the risk of chronic diseases due to its potential antioxidant and anti-inflammatory activities [23, 24]. In addition, kaempferol displays chemopreventive activity by inhibiting cancer cell growth and angiogenesis and by inducing cancer cell apoptosis [23]. Our investigation showed that kaempferol demoted airway eosinophil infiltration and subepithelial fibrosis in endotoxin-exposed airway epithelial cells and in asthmatic mice [26, 27]. Oral administration of kaempferol deterred OVA inhalation-induced loss of epithelial morphology and increase of collagen deposition in mouse lung tissues [27]. Also, this polyphenol suppressed epithelial excrescency and goblet hyperplasia observed in the lung of OVA-challenged mice [27]. Based on our previous findings, the present study explored that kaempferol inhibited mucus hypersecretion linked to goblet cell hyperplasia triggered by TGF-β. The inhibitory effects of kaempferol on airway mucus hypersecretion in asthma are not well defined. Therefore, this study explored the inhibitory mechanism(s) by which kaempferol encumbered the MUC5AC induction in TGF-β-exposed airway epithelial cells and in OVA-sensitized mice.

High expression of TGF-β plays an essential role in airway remodeling and correlates with subepithelial fibrosis [31, 32]. Blocking TGF-β activity dampens epithelial shedding, mucus hypersecretion, angiogenesis, hypertrophy and hyperplasia of airway smooth muscle cells in an asthmatic mouse model [32, 33]. In our previous study, LPS induced TGF-β signaling in airway epithelial cells through enhancing the expression induction of TGF-β1 and its receptors, and the OVA challenge highly induced TGF-β in mouse lung tissues [27]. Nevertheless, the cellular origin of this cytokine in the asthmatic airways is still a contemporaneous debate [34]. In the current study, TGF-β and OVA enhanced the muc5AC induction in airway epithelial cells and lung tissues, respectively. The OVA inhalation culminated the increase in goblet cell proliferation. Since goblet cells are responsible for the production of mucus, the increased growth and stimulation of goblet cells results in an increase in mucus production and secretion [33, 35]. It can be assumed that the OVA induction of TGF-β activity promoted goblet cell hyperplasia and mucus hypersecretion in asthmatic airways.

ER stress and UPR activation are cardinal modulators of inflammatory responses in the pathogenesis of emphysema [36]. ER stress responses inhibit subepithelial fibrosis associated with loss of lung function, indicating that their inhibition may provide a potential therapeutic avenue in chronic allergic airways disease [20]. One investigation shows that house dust mite provokes ER stress in airway epithelial cells by enhancing the induction of ATF6α and ERp57 protein disulfide isomerase [20]. The current study showed that like tunicamycin TGF-β and OVA stimulated ER stress through increasing the induction of UPR components and molecular chaperones of IRE1α and GRP78 and the splicing of XBP-1 mRNA. Chemical chaperones such as glycerol, trehalose, and trimethylamine-N-oxide reduced UPR component markers, airway inflammation, and asthmatic remodeling [19]. In this study, kaempferol mitigated the UPR responses leading to ER stress triggered by tunicamycin, TGF-β and OVA. Accordingly, these results indicate a crucial role of the ER stress in the pathogenesis of OVA-associated allergic asthma and show therapeutic potential for the polyphenol kaempferol like chemical chaperones.

Protein misfolding and ER stress are observed in intestinal secretory cells from mouse models, and the UPR activation induces intestinal inflammation [37]. This study attempted to explore that the mucus hypersecretion entailed the induction of ER stress due to the allergen exposure in bronchial airways. The ER stressor tunicamycin enhanced the induction of MUC5AC protein linked to the bronchial mucus hypersecretion, which was blocked by kaempferol. It should be noted that the induction of the UPR markers occurred within 24 h after the stimulation of epithelial cells with TGF-β or tunicamycin, and the MUC5AC protein was highly induced by its 72 h-stimulation. These results indicate that the mucus hypersecretion may be attributed to the UPR responses following the ER stress of airway goblet cells. Consistently, the goblet cell dysplasia and the XBP-1 induction were compatibly observed in the pulmonary tracts of OVA-experienced mice. ER stress of human LS174T goblet cells with tunicamycin and the protein misfolding of MUC2 in Winnie mice result in the production of intestinal mucus, and the inhibition of the mucin production by interleukin (IL)-10 helps the intestine preserve the mucus barrier [38].

This study investigated the inhibitory mechanisms of mucin induction by kaempferol in bronchial airway cells during the ER stress responses. The loss of ER stress protein anterior gradient homolog 2 reduced overproduction of MUC5AC and MUC5B in individuals with asthma and in mouse models of allergic airway disease [39]. IRE1β is shown to be functionally required for airway mucin production and is a potential mucous cell-specific therapeutic target for airway diseases [40]. In this study kaempferol inhibited the MUC5AC induction through disturbing RE1α-XBP-1-mediated UPR signaling cascades of TRAF2 and JNK in TGF-β-exposed bronchial epithelial cells and OVA-challenged lung tissues. Accordingly, kaempferol can be considered as anti-hypersecretory agent in the health and diseases in airways and lung. Other investigation has shown that 6-gingerol, an active polyphenol present in ginger, suppresses the expression of IL-1β-induced MUC5AC mRNA and protein in human airway epithelial cells via blocking ERK- and p38 MAPK-responsive pathways [41]. In addition, the green tea polyphenol (-)-epigallocatechin-3-gallate markedly inhibits IL-1β-induced MUC5AC gene expression and MUC5AC secretion through suppressing activation of ERK, mitogen- and stress-activated Kinase 1, and cAMP response element-binding protein [42]. Furthermore, several studies reveal that flavonoids regulate MUC5AC production via regulating a mechanism involving nuclear factor-κB pathway [43, 44]. There are few studies dealing with therapeutic natural compounds that antagonize mucus hypersecretion in pulmonary tracts following the ER stress of airway secretory cells.

In summary, this study investigated the potential of kaempferol as a target for therapeutic strategies in alleviating goblet cell hyperplasia and congruent mucus hypersecretion linked to the induction of MUC5AC protein. Furthermore, TGF-β and OVA inhalation instigated ER stress through enhancing IRE-1α phosphorylation and XBP-1 splicing in epithelial cells. Kaempferol deterred the TGF-β- and OVA-prompt ER stress leading to mucin induction in airway tissues. ER stress involving TGF-β signaling entailed TRAF2 induction and JNK activation responsible for mucin production. Therefore, kaempferol was effective in ameliorating mucus hypersecretion through disturbing TGF-β-triggered ER stress signaling of IRE1α-TRAF2-JNK in cellular or animal models of allergic asthma.
